# Surgeons’ views on preoperative medical evaluation: a qualitative study

**DOI:** 10.1186/s13741-017-0072-5

**Published:** 2017-10-24

**Authors:** Kevin R. Riggs, Zackary D. Berger, Martin A. Makary, Eric B. Bass, Geetanjali Chander

**Affiliations:** 1 Division of Preventive Medicine, University of Alabama at Birmingham, Birmingham, AL USA; 2 Division of General Internal Medicine, Johns Hopkins University School of Medicine, 1830 E. Monument Street, Room 8060, Baltimore, MD 21287 USA; 3 Department of Surgery, Johns Hopkins University School of Medicine, Baltimore, MD USA

**Keywords:** Preoperative care, Risk assessment, Medical overuse

## Abstract

**Background:**

There is substantial variation in the practice of preoperative medical evaluation (PME) and limited evidence for its benefit, which raises concerns about overuse. Surgeons have a unique role in this multidisciplinary practice. The objective of this qualitative study was to explore surgeons’ practices and their beliefs about PME.

**Methods:**

We conducted of semi-structured interviews with 18 surgeons in Baltimore, Maryland. Surgeons were purposively sampled to maximize diversity in terms of practice type (academic vs. private practice), surgical specialty, gender, and experience level. General topics included surgeons’ current PME practices, perceived benefits and harms of PME, the surgical risk assessment, and potential improvements and barriers to change. Interviews were audio-recorded and transcribed. Transcripts were analyzed using content analysis to identify themes, which are presented as assertions. Transcripts were re-analyzed to identify supporting and opposing instances of each assertion.

**Results:**

A total of 15 themes emerged. There was wide variation in surgeons’ described PME practices. Surgeons believed that PME improves surgical outcomes, but not all patients benefit. Surgeons were cognizant of the financial cost of the current system and the potential inconvenience that additional tests and office visits pose to patients. Surgeons believed that PME has minimal to no risk and that a normal PME is reassuring to them and patients. Surgeons were confident in their ability to assess surgical risk, and risk assessment by non-surgeons rarely affected their surgical decision-making. Hospital and anesthesiology requirements were a major driver of surgeons’ PME practices. Surgeons did not receive much training on PME but perceived their practices to be similar to their colleagues. Surgeons believed that PME provides malpractice protection, welcomed standardization, and perceived there to be inadequate evidence to significantly change their current practice.

**Conclusions:**

Views of surgeons should be considered in future research on and reforms to the PME process.

## Background

Patients often undergo extensive, multidisciplinary evaluation prior to elective surgery. Initially, the evaluation is aimed at determining whether patients indeed have a surgical condition. Once this has been determined and a tentative decision to proceed with surgery has been made, patients often undergo additional preoperative medical evaluation (PME)—testing and evaluation aimed at assessing and minimizing surgical risk (Riggs and Segal 2016). There is substantial variation in this practice of PME (Thilen et al. 2013; van Gelder et al. 2012; Wijeysundera et al. 2012) and limited evidence for benefit (Balk et al. 2014; Wijeysundera et al. 2010), which has raised concerns about overuse (Baxi and Lakin 2015; Brateanu and Rothberg 2015; Smetana 2015).

The perioperative process is complex and includes diverse provider types, including surgeons, anesthesiologists, primary care and medical specialists, and spans multiple settings, including the outpatient clinic, operating room, and hospital. Surgeons are uniquely situated as they follow patients through the entirety of this process, from prior to surgery to the hospital to the postoperative follow-up. This unique vantage point may give surgeons important insights regarding the processes of PME that can assist with improving the efficiency of the system. However, knowledge of surgeons’ views on PME is limited. Several recent qualitative studies focused narrowly on preoperative testing in low-risk situations (Brown and Brown 2011; Patey et al. 2012), and several quantitative surveys focused on preoperative consultations (Katz et al. 1998; Pausjenssen et al. 2008). The objective of this study was to more broadly explore surgeons’ modern practices and beliefs about PME.

## Methods

### Study design

We conducted a qualitative study consisting of semi-structured interviews with surgeons. Because the preoperative process is complex and so little is known about surgeons’ practices and what motivates those practices, we felt that a qualitative approach would allow us to explore that complexity better than a quantitative survey.

### Setting and participants

Surgeons were recruited from clinical practices in Baltimore, Maryland. We purposively recruited surgeons to maximize the diversity of our sample with the goal of hearing a wide range of practices and opinions. We recruited an approximately equal mix of academic and private practice surgeons, and a mix of general (including colorectal, vascular, oncologic, thoracic, endocrine, breast, and plastic) and non-general (including orthopedic, urologic, and otolaryngologic) surgical specialties. Additionally, we oversampled women and sought a mix of early-, mid-, and late-career surgeons.

We contacted surgeons from two local academic institutions via publically available email addresses. We were unable to locate publically available email addresses for local private practice surgeons, so we obtained their contact information with the assistance of the Johns Hopkins Clinical Research Network, a research consortium of area hospitals and health systems. All surgeons were located in and around Baltimore, Maryland. Surgeons were offered a small monetary incentive for participating, and they were interviewed in-person in a private setting that was convenient for them, typically their offices.

### Interview guide

General topics for questioning included surgeons’ current PME practices and related training, surgical risk assessment, potential benefits and potential downsides for patients and others in the health care system, and ideas for improving the system. Each author was involved in developing the initial interview guide. As the study progressed, the interview guide underwent revisions to allow further exploration of new topics that had been raised in prior interviews. Additionally, semi-structured interviews allowed for topics not included in the guide to emerge and be explored. The final interview guide is shown in the Appendix.

### Data collection and analysis

One investigator (KRR) conducted the interviews in person between June 2015 and May 2016. Just prior to the interview, participant characteristics were collected using a brief questionnaire. Interviews were audio-recorded and the recordings were transcribed verbatim. Identifying information was removed from interview transcripts.

The transcripts were initially analyzed using conventional thematic content analysis (Hsieh and Shannon 2005). A codebook of descriptive codes (also known as topical codes) was developed collaboratively by two authors (KRR and GC) as the transcripts were reviewed. The transcripts were coded using textual data analysis software (ATLAS.ti version 7, Scientific Software Development), which allowed coded segments to be compared to all other segments with the same code to look for emerging themes. The outcome of this type of thematic analysis is the identification of general themes (e.g., reassurance as a benefit of preoperative medical evaluation), which can then be used to construct new theory (e.g., as with grounded theory methodologies). The goal of our analysis was not to develop theory but to identify themes in the form of specific assertions representing practices and beliefs (Erickson 1986; Saldaña 2013). The initial stage of coding was ongoing during the time interviews were being conducted, and helped guide the determination that enough data had been collected (a concept known as thematic saturation) (Guest et al. 2006).

After the initial stage of thematic analysis, each of the transcripts was then re-coded for supporting and opposing instances of each theme (except for “practice variation” which we just described). We tabulated the number of interviews in which instances of each theme appeared (not necessarily mutually exclusive, as supporting and opposing instances of a theme could each appear in an interview). Each transcript was independently coded by two team members to enhance reliability, and discrepancies were resolved by consensus. The institutional review board at the Johns Hopkins University School of Medicine approved the study.

## Results

We interviewed a total of 18 surgeons, whose demographic characteristics are shown in Table 1. One-third of the surgeons were female, and the mean age and mean time since finishing their training was 43.4 years and 9.7 years, respectively. Surgeons were evenly split between academic and private practice, and slightly more than half (11/18) were general surgery specialties.

**Table 1:** Surgeon characteristics

	(*n* = 18)
Gender, female	6
Age, mean (range)	43.4 (32–66)
Years since completed training, mean (range)	9.7 (1–36)
Practice setting	
Academic	9
Private practice	9
Surgery type	
General	
Colorectal	4
Vascular	2
Oncologic	1
Thoracic	1
Endocrine	1
Breast	1
Plastic	1
Non-general	
Orthopedic	5
Urologic	1
Otolaryngologic	1

### Practice variation

The variation in surgeons’ descriptions of their PME practices was striking. Each surgeon said they required an evaluation and some testing for a majority of their patients, typically directed by primary care physicians (PCPs) or preoperative clinics run by anesthesiology. However, some surgeons were selective about who would require these additional visits based on patient factors and operation factors, while others required these visits in all of their patients with no exceptions. Surgeons were split on whether they preferred PCPs or anesthesiologists direct the PME, and while most surgeons felt that either was adequate, some required patients to be seen separately by both. Some surgeons ordered necessary tests themselves, while others had consultants order them (sometimes at the discretion of the consultants, and other times dictated by the surgeons). Some surgeons required specialist involvement (e.g., cardiology) in certain cases, while others left that determination to the discretion of the clinician performing the PME. Some surgeons would require visits with several specialists in addition to a PCP visit for some patients, while others said that a specialist visit should obviate the need for a PCP visit. Most surgeons initiated the process of evaluation after the tentative decision for surgery had been made, and they added patients to the operating room schedule at that point. However, a few surgeons said they waited for the results of preoperative consults or tests before adding patients to the operating room schedule to make sure they were “cleared.”

### Patient benefits and harms

Themes related to the benefits and harms of PME and representative quotations are shown in Table 2. Universally, surgeons believed that PME provided some benefit to at least some patients. The primary benefit that surgeons cited was the identification of occult conditions. Many shared stories of patients who appeared well but had serious pathology incidentally discovered in the PME, which was either able to be addressed (making surgery safer) or required surgery to be canceled (saving the patient an unnecessary or unsafe surgery). Surgeons also indicated that they believed the PME resulted in optimization or “fine tuning” of chronic conditions, although they were less specific in their evidence supporting this belief.

**Table 2:** Themes related to benefits and harms of preoperative medical evaluation

Theme	Number of interviews supporting theme, representative quote	Number of interviews opposing theme, representative quote
The preoperative medical evaluation can improve surgical outcomes by identifying treatable occult conditions and/or optimizing chronic conditions.	12 interviews “I think it prevents catastrophes. I mean, you wouldn’t want to carry someone to the operating room and have the stress test on the operating room table. But by doing a preoperative evaluation you might uncover, whether it’s the EKG, whether it’s laboratory findings, you might uncover something that suggests this person is at high risk for cardiovascular disease which might lead to other evaluation which could prevent a misadventure, so to speak, in the operating room.” -Academic urologic surgeon	2 interviews “I mean if you believe the trials, you know, the CARP trial being the classic one, there is no benefit for preoperative evaluation.” -Academic vascular surgeon
Many patients do not medically benefit from the preoperative medical evaluation	8 interviews “How many pre-ops do you have to do before you find something really wrong? Because most people, you are not going to find anything in that kind of situation.” -Private practice colorectal surgeon	0 interviews
A normal preoperative medical evaluation before surgery is reassuring to patients and physicians	6 interviews “There is a peace of mind I get from it, because if I have those numbers and objective data in front of me then it makes me feel more confident that the patient is going to be safe.” -Academic endocrine surgeon	0 interviews
The preoperative medical evaluation has minimal to no medical risk for patients	12 interviews “I don’t see an overt downside to a preoperative evaluation other than the logistics of it, frankly.” -Academic vascular surgeon	2 interviews “It’s sort of like screening for most conditions, where if they’re not symptomatic and you find something, you are forced- you’re not forced, but the standard of care at that point is to pursue it, and sometimes that even postpones surgery because something has been discovered that may or may not be clinically important, and so I think it’s just a lot of extra engagement with the health care system.” -Academic otolaryngologic surgeon
Additional office visits and tests can be an inconvenience for patients	16 interviews “It’s cumbersome. I mean it’s difficult. They have to come back on a separate day. A lot of my out-of-towners, it’s difficult, right, because let’s say you live in Kansas and you are coming here for surgery and you see me and we schedule surgery. You go home and see your family, and now you’ve got to come back a couple of days before the surgery to go to the [preoperative clinic]. And so it’s an inconvenience for sure.” -Academic oncologic surgeon	1 interviews “But, interestingly enough, the patients accept it very willingly. Almost expected, not just accepted.” -Academic colorectal surgeon
The financial cost to the patient and/or healthcare system is a downside of the preoperative medical evaluation.	14 interviews “It’s expensive and most of it is not necessary. And it’s expensive both from the standpoint of true monetary cost of doing testing but also there’s a time expense for everybody.” -Academic endocrine surgeon	3 interviews “And I think there is a benefit in that if you can prevent a complication, there is a huge benefit not only clinically, but also financially.” -Academic oncologic surgeon

Surgeons generally agreed that PME was overall beneficial, though many acknowledged that the benefits typically accrue to a minority of patients and that most patients did not derive a direct medical benefit. Some surgeons pointed out that it is not possible to identify who will benefit ahead of time, and so PME that ends up not being beneficial is ultimately justified by the minority of cases where patients derive a medical benefit.

Even when the results of the PME are normal and do not alter the surgical plan, some surgeons believed that they were still worthwhile. In some cases, this was just because the surgeons like to “have those numbers.” In addition, they believed that normal test results and “another pair of eyes” reviewing the case were reassuring to patients as well as surgeons and anesthesiologists. Other potential patient benefits were mentioned less frequently, including as opportunities for education or to increase patient engagement, or just a reason for people who might not otherwise see a primary care doctor to do so.

The majority of surgeons denied that there could possibly be any medical harms from PME. While many of the surgeons explicitly recognized that PME essentially represents “screening” that patients may not otherwise receive if they were not considering surgery, they believed that anything identified through this screening (e.g., lab abnormalities or lung nodules) ultimately represented a benefit to patients. However, one surgeon cited the potential of falsely abnormal results to delay surgery and another specifically cited “overtreatment.”

Surgeons generally downplayed potential medical harms of the PME, but they largely recognized the inconvenience to patients of extra office visits and tests. Some mentioned that visits in preoperative clinics the day before surgery would require out-of-town patients to spend a night in a hotel. A number of surgeons mentioned that patients had brought up the inconvenience of the PME to them, although only one had ever heard a patient bring up out-of-pocket costs for extra office visits or tests.

### Surgical risk assessment

Themes related to risk assessment and representative quotations are shown in Table 3. Universally, surgeons indicated that in their initial evaluation of patients, they performed their own assessment of patients’ surgical risk. For the most part, these assessments were informal, described as “a gestalt,” “the eyeball test,” or “spit balling.” Few had ever used a formal risk assessment tool or calculator, and none reported using them regularly. However, surgeons were generally confident in their ability to estimate risk. Two surgeons mentioned research indicating that surgeons’ gestalt is as good as a more formal risk assessment, and one even said that if their assessment differed from the consultant’s assessment that “I still trust myself more.”

**Table 3:** Themes related to risk assessment

Theme	Number of interviews supporting theme, representative quote	Number of interviews opposing theme, representative quote
Surgeons are confident in their ability to assess patients’ surgical risk.	13 interviews “I think we get a good gestalt of the patients overall by the time you get through the H&P. The longest assessment is the initial visit. Once you can confirm their medical history and the medications you get a good sense of their risk.” -Academic orthopedic surgeon	5 interviews “I don’t really have the expertise to figure out their cardiac risk whereas the other providers generally do… I mean, qualitatively I can tell. Some patients are probably going to be higher risk than others, but beyond that I’m not really trained in what to specifically look for.” -Private practice orthopedic surgeon
The risk assessment provided by non-surgeons is viewed primarily as either clearing or not clearing the patient for surgery.	11 interviews “I’ll generally make sure there’s no glaringly abnormal laboratory studies and make sure at least the note from PCP says the patient is cleared for surgery or moderate—make sure it doesn’t say they’re at extremely high risk or really not cleared for surgery. If that were the case, then they probably would have notified me beforehand, so I’m not going to read [the note] in detail.” -Private practice orthopedic surgeon	4 interviews “If I was about to do a big operation on someone and the preop says, “whoa, whoa, whoa, hold on, this is a really sick person,” sometimes there’s a lesser option. If they uncover things that I wasn’t aware of, I might go with, for obstructing colon cancer, I might go with a stoma. Just give them a bag, a quick operation, rather than trying to take it out and do this five hour operation.” -Academic colorectal surgeon

Some surgeons found the risk assessment provided by the consultants performing the PME to be helpful, but most reported that it rarely affected their surgical plan. Most surgeons viewed the PME a task that had to be completed, and a majority of surgeons spontaneously used the terminology “cleared” or “clearance” to describe the assessment of the clinicians performing PME, reflecting this belief. This view is driven in part by the timing of the PME, which typically occurs after a tentative decision to have surgery has been made, so surgeons have already made an assessment that the benefits outweigh the risks. To this end, some surgeons explicitly said that they do not routinely review the notes provided by consulting clinicians, and that when there are problems that are going to delay or prevent surgery, they are contacted directly about them. So while other clinicians’ risk assessment is not routinely incorporated in the decision to have surgery, unanticipated problems discovered during the PME often result in the surgery being reconsidered.

### Drivers of current PME practice, potential improvements, and barriers to change

Themes related to drivers of current PME practice, potential improvements, and barriers to change and representative quotations are shown in Table 4. Most surgeons indicated that their requirements for preoperative testing and medical office visits were driven by hospital or anesthesiology requirements, although they were not always able to describe the specifics of those requirements. Many surgeons were focused on ensuring that anesthesiology’s requirements were met to ensure that cases were not canceled at the last minute, sometimes driving surgeons to order or request more than they thought was actually needed.

**Table 4:** Themes related to drivers of current practice, potential improvements and barriers to change

Theme	Number of interviews supporting theme, representative quote	Number of interviews opposing theme, representative quote
Hospital and/or anesthesiology requirements (including informal or perceived requirements) are a major driver of surgeons’ use of preoperative services.	14 interviews “For major surgery, everybody needs to get a set of pre-operative tests, and the standards are set mostly by the hospital where the surgery is going to take place. Some of these hospitals send us a grid and on one side there are patient’s characteristics like age, whether they are a smoker, whether they have certain medical conditions. On the other end, based on the response to the first set of questions, it tells us whether we should get an electrocardiogram, a chest X-ray, some blood work, etc. What we do most of the time, because it’s always better to have more than have your surgeries get delayed because you didn’t get enough, we go ahead and get more extensive blood work. Let’s say they request basic chemistry, we may go ahead and get some liver function tests as well.” -Private practice colorectal surgeon	2 interviews “I get coags on everybody and I think that some people push back and say you don’t need to do that unless somebody has some history of a bleeding problem or something along those lines. [Even if anesthesia didn’t require them], I would still get them.” -Academic orthopedic surgeon
Surgeons receive minimal formal training on performing preoperative medical evaluations.	13 interviews You know, my residency didn’t really—we do kind of general surgery time and stuff like that, but certainly in medical school, I never really learned “here’s how to properly preop the patient.” We sort of encountered it a lot as you would, for example, consult medicine to see if you can fix some old lady’s hip. You’d sort of learn indirectly by seeing how they would clear them, but I guess I never really learned formally the right ways to do it. -Private practice orthopedic surgeon	1 interview I did [get specific training about what to order on who and why] for disease processes that could produce intraoperative problems. So hyperthyroidism, pheochromocytoma, those sorts of things. Did I get training about more subtle things? Like, for example, this person is taking Plavix, here are the things you need to do about those patients. Not really. -Academic endocrine surgeon
Surgeons’ preoperative medical evaluation practices are similar to their colleagues’ practices.	14 interviews “My senior partners had been doing this for 30 years, and so I kind of just picked up the flow of how the office works and how they do [the preoperative medical evaluation].” -Private practice breast surgeon	4 interviews “I think my colleague probably does fewer testings, like in terms of cardiac clearance. I think that he may, for example, instead of having a cardiology work them up, he says, I’ll just go ahead and order an ECHO or a stress test. And if those look fine then he clears them.” -Private practice thoracic surgeon
The preoperative medical evaluation reduces surgeons’ malpractice risk.	12 interviews “I guess it probably would give you a little defense if patient develop post-op medical issue. Then it probably will be a help defensively to say hey, I got the medical opinion on that. There’s no question that’s part of the deal when you get a medical clearance.” -Private practice vascular surgeon	2 interviews “I guess it could go both ways in the sense that I mean in theory [the preoperative medical evaluation] should lower [the malpractice risk], but in reality if you do not read the note, it may increase it.” -Private practice colorectal surgeon
Surgeons welcome standardization of preoperative medical evaluation protocols.	6 interviews “I think there were sort of national or at least sort of acceptable agreed upon standards between institutions, I think that would just be a lot easier because then theoretically it would be more interchangeable. Like if we just agreed that, you know, this is the definitive article and here’s this grid. Wherever you fall on this grid, this is how we’ve decided as a medical community that people are cleared for surgery. Then you can go anywhere and do it and you know what people are going to get, and it kind of takes the guesswork out.” -Private practice orthopedic surgeon	2 interviews “It would be nicer if things weren’t mandatory, because in some cases--especially in the hospital--if a patient is coming in two months later, and their pre-op was done 75 days ahead, and you know that there hasn’t been a whole lot of change in the interim, it would be nice if we could kind of use our judgment. But, they don’t allow room for that, because I guess they don’t want to trust people’s judgment.” -Private practice plastic surgeon
There is inadequate evidence regarding the benefits of preoperative medical evaluation.	3 interviews I definitely don’t know if it is absolutely necessary to preop my young 20-year olds. I mean is there a possibility that something could pop up during some of their routine blood work or something? Of course. But you know that from a cost analysis standpoint I don’t know if it is really needed. More studies that need to look into that. -Private practice orthopedic surgeon	0 interviews

Most surgeons reported receiving very little formal training in how to perform the PME. Some of the general surgeons indicated that during their training, they were responsible for performing PME, so they gained some expertise and comfort in doing so, even if they had to “muddle through and figure it out.” On the other hand, most of the specialty surgeons indicated that they outsourced PME to primary care or anesthesia during their training, and so they never gained any experience or comfort with it.

Despite the variation and lack of training, most surgeons perceived that their practice was very similar to their colleagues’. In part, this related to hospital requirements that essentially standardized both their practice and their colleagues’ practice. However, several explicitly stated that they developed their current practice by observing and adopting that of their colleagues.

Many surgeons felt the PME reduced their malpractice risk, although this was not universal. Some indicated that the reduced risk was due to the PME leading to fewer complications, but many indicated that even in the case of non-preventable complications, the fact that another physician had “cleared” the patient for surgery would be protective.

Several surgeons indicated that they welcomed more standardization of the PME. Toward this end, several thought consensus guidelines would be helpful because they would reduce their uncertainty as to whether the patient had received an adequate PME and may even reduce the malpractice risk. However, several surgeons expressed skepticism that there was adequate evidence that a less resource-intensive PME process would be safe, although they indicated that more evidence could persuade them to change their practice.

## Discussion

In this qualitative study, we elicited a comprehensive picture of surgeons’ views on PME. This study supplements prior studies, which were more narrowly focused either on testing in low-risk situations (Brown and Brown 2011; Patey et al. 2012) or medical consultations (Katz et al. 1998; Pausjenssen et al. 2008). For example, Brown and Brown (Brown and Brown 2011) conducted qualitative interviews with a diverse group of clinicians involved with preoperative testing (which included 7 surgeons out of 23 participants), and reported themes similar to ours with respect to satisfying hospitals’ and anesthesiologists’ requirements to avoid delays and cancellations, the potential medicolegal benefit of preoperative testing, and clinicians’ desire to have more standardization. However, most of our themes were not reported in these previous studies.

The results of this study have several important implications for future efforts to improve the process of PME. First, the PME should not primarily be viewed as simply providing a “risk assessment” or “risk stratification.” Surgeons are generally confident in their ability to estimate surgical risk, and since a tentative decision to proceed with surgery has already been made, simply providing another risk estimation will not routinely cause those decisions to be reconsidered. Rather, surgeons are looking for confirmation of their assessment (i.e., that they did not miss something important). Surgeons also want to assure that patients’ chronic conditions are being appropriately treated (i.e., “optimization” (Riggs and Segal 2016)), although how often conditions are actively being managed during this process is uncertain. Additionally, more research on who is best suited to direct the PME (i.e., PCPs or dedicated preoperative clinics) may be helpful, though as described by several surgeons in the study, it is likely unnecessarily redundant to have more than a single preoperative assessment.

Second, surgeons recognize that not every patient benefits from the PME, but they do believe that the process improves surgical outcomes overall. Further, while they are aware of the potential inconvenience of the process for patients, they do not believe that current evidence is adequate to justify changing their practice to require fewer tests or office visits. While some commentators have argued that evidence is sufficient to limit preoperative testing in many situations (Brateanu and Rothberg, 2015; Smetana 2015), high-quality evidence demonstrating the safety of less intensive PME is generally lacking (Balk et al. 2014) (with the exception of cataract surgery (Schein et al. 2000)). High-quality evidence demonstrating the safety of forgoing certain preoperative services and research examining the effect of less intensive PME on patient experience has the potential to change practices.

Finally, surgeons were very clear that one of their primary concerns is satisfying anesthesia and hospital requirements in order to avoid last minute cancellations. In part, this problem arises from the current workflow, where anesthesiologists may be assigned to cases on the day before or the day of surgery. Variation in the anesthesiologists’ opinion of what constitutes an appropriate PME may drive surgeons to be more exhaustive in terms of preoperative tests and consultations than they otherwise would be, in order to avoid last-minute delays and cancellations. More uniformity about what anesthesiologists expect would be welcomed by surgeons, as it would decrease anxiety about delays and cancellations and may lead to a less intensive PME. Consensus guidelines, even in the absence of more high-quality evidence, could drive more standardization and could even allay surgeons’ concerns about malpractice liability (Kirkpatrick and Burkman 2010). However, surgeons seem more attentive to local hospital policies than to national clinical guidelines, so future guidelines would likely have more impact if targeted at hospitals and anesthesiologists rather than surgeons or internists who perform PME.

This study has several limitations. First, we presented our themes as assertions, although qualitative research is not meant to be hypothesis testing, so these assertions warrant further quantitative testing in more representative samples. Second, all surgeons were currently practicing in a small geographic region, so their practices and beliefs could be specific to that region. Finally, we tried to avoid any specific focus on overuse or low-value care, though surgeons may have perceived that to be an implicit subject and tailored their interviews to avoid describing some low-value practices.

## Conclusion

Surgeons’ PME practices vary dramatically, even within a single geographic area. While overuse in PME may be a legitimate concern, surgeons generally view PME as beneficial, so future research on PME and future reforms to the PME process should take into account surgeons’ views on the topic. More research into the safety of forgoing certain preoperative services and patient satisfaction with less intensive PME, and increasing standardization of the process through consensus guidelines may be options to decrease unwarranted variation and increase the value of PME.

## Appendix

### Final interview guide

1. What are the operations you most commonly perform?
2. What is your typical patient population like?
3. What is your standard practice for “preops”? (Or whichever term is familiar.)
4. Who usually performs those preops?
5. What tests do you recommend or require be obtained as part of the preop?
6. Do you order those tests? Why or why not?
7. Do you request specialists to be involved in the preop? If so, how do you make that decision?
8. How do you request a preop?
9. How do you process preop notes and test results?
10. Is there something specific in the preop note that you are looking for?
11. (If not everyone gets a pre-op) How do you decide who doesn’t need a pre-op?
12. Does your practice or institution have rules about preops? If so, what are they?
13. Are there any clinical guidelines for preops that affect your practice? If so, what are they?
14. How were you trained about doing preops?
15. How does your current practice differ from the way you were trained?
16. How do you think your practice differs from your colleagues? Others in your community?
17. In your opinion, what are the benefits to the patients from preops?
18. What are the potential downsides for the patients?
19. Do you ever change your surgical plan based on the preop? If so, how?
20. What are the benefits to you, the other providers involved, or the health system from preops? How do preops affects a surgeon’s risk of malpractice?
21. What are the potential downsides to you or the health system from preops?
22. Do anesthesiologists ever cancel cases because of inadequate preop? If so, examples?
23. How comfortable do you feel in estimating a patient’s operative risk?
24. How do you estimate a patient’s operative risk?
25. Do patients express preferences or expectations regarding preops? If so, how?
26. Do you have any memorable anecdotes about a patient who was impacted positively or negatively by a preop?
27. Do you think there would be any way to make the preop system better?
28. Anything else?

## Abbreviations

PCP: Primary care physician
PME: Preoperative medical evaluation
